# Use of Complementary and Alternative Medicine among Patients with End-Stage Renal Disease

**DOI:** 10.1155/2013/654109

**Published:** 2013-04-23

**Authors:** Gurjeet S. Birdee, Russell S. Phillips, Robert S. Brown

**Affiliations:** ^1^Division of General Internal Medicine & Pediatrics, Department of Medicine, Vanderbilt University Medical Center, Nashville, TN 37232, USA; ^2^Vanderbilt Center for Integrative Health, Vanderbilt Center for Health Services Research, Vanderbilt University Medical Center, Suite 6000 Medical Center East, Nashville, TN 37232-8300, USA; ^3^Division of General Medicine and Primary Care, Beth Israel Deaconess Medical Center, Boston, MA 02215, USA; ^4^Division of Nephrology, Department of Medicine, Beth Israel Deaconess Medical Center, Boston, MA 02215, USA

## Abstract

Among patients with end-stage renal disease (ESRD), few studies have examined the use of complementary and alternative medicine (CAM) and patients' interest in learning mind-body interventions to address health issues. We surveyed 89 adult patients (response rate 84%) at an outpatient hemodialysis center in Brookline, MA, USA regarding the utilization of CAM, including mind-body practices, and willingness to learn mind-body practices. Of respondents, 47% were female, 63% were black, and mean age was 62 years. 61% reported using CAM for health in their lifetime, and 36% reported using CAM within a month of the survey. The most frequent CAM modalities reported in ones' lifetime and in the last month were mind-body practices (42% and 27%, resp.). Overall lifetime CAM use did not differ significantly by sex, race, dialysis vintage, diagnosis of ESRD, employment status, or education level. Subjects reported that mind-body interactions were very important to health with a median score of 9 on a 10-point Likert scale (ranging from 0 for not important to 10 for extremely important). Most patients (74%) reported interest in learning mind-body practices during maintenance hemodialysis. In summary, CAM use, particularly mind-body practice, is frequent among patients with ESRD providing opportunities for future clinical research.

## 1. Introduction

Over half a million individuals in the USA have end-stage renal disease (ESRD), and about 400,000 receive maintenance hemodialysis (MHD) to sustain their life [1]. Despite significant medical advances over several decades, the survival of patients on MHD remains drastically shorter than the general population with a reported 5-year survival rate of 35% in the USA [1]. Cardiovascular diseases account for 50% of mortality [1], in part due to a disproportionate burden of cardiovascular risk factors such as hypertension, diabetes, dyslipidemia, and sedentary behavior [2]. Patients receiving MHD also experience lower health-related quality of life [3, 4] and physical functioning [5–7] as compared to healthy individuals with normal kidney function and suffer from a high prevalence of mood disorders [8, 9]. A majority of research on improving health outcomes for patients on MHD has focused on pharmacotherapy and delivery of MHD [10], with less research in the area of behavioral interventions such as exercise [11–14]. While initial research suggests that exercise may improve health-related outcomes among patients with ESRD [15, 16], implementation has been challenging [12, 17]. Investigators have studied exercise prescription outside of and during dialysis sessions, and intradialysis exercise may have a higher adherence than extradialysis exercise [15, 18]. 

 Complementary and alternative medicine (CAM) may provide new therapeutic options for patients with ESRD with the goal of improving symptoms and quality of life. CAM, as defined by the National Center for Complementary and Alternative Medicine, is “a group of diverse medical and health care systems, practices, and products that are not generally considered to be a part of conventional medicine” [19]. Based on national survey data, four out of 10 adults in the USA have used some type of CAM in the last 12 months [20]. The most commonly used CAM modalities include biologically based products (herbs and dietary supplements) and mind-body practices (e.g., yoga, tai chi, deep breathing, and meditation). However, there are limited data on the use of or interest in CAM therapies among patients with chronic kidney disease including those with ESRD [21–23]. In particular, mind-body practices may provide a novel behavioral treatment for patients on MHD to reduce comorbid symptoms and conditions associated with this complex chronic disease. Similar to exercise, mind-body intervention could potentially be delivered during MHD; though patients' willingness to practice mind-body techniques is unknown. The purpose of this study among patients with ESRD on MHD was to identify the frequency of prior CAM use, including mind-body practices, attitudes towards mind-body practices, and willingness to learn or participate in studies of mind-body practices during MHD.

## 2. Methods

### 2.1. Study Population

All patients with ESRD receiving MHD at a private dialysis center in Brookline, MA, USA, affiliated with a large academic medical center from September 2009 to July 2010 were approached to participate in the study. Potentially eligible patients were approached chair side during their dialysis session and asked to participate in the study. Subjects were excluded from the study if they were younger than 18 years, non-English speaking, or had a diagnosis of dementia. Willing patients gave verbal consent to participate. 

### 2.2. Survey Instrument and Data Collection

A 40-item questionnaire was administered by the principal investigator (GB) to subjects face-to-face, chair side, during MHD (see Table 1 complementary and alternative medicine questionnaire). Questionnaire items were adapted from National Health Interview Survey Adult Complementary and Alternative Medicine Supplement, a nationally representative survey administered by the CDC in 2007 [20]. Items were selected based on content to determine the prevalence of CAM use in the subject's lifetime and the last month. In addition, we asked questions to explore patients' perception of mind-body practices including importance of mind-body interactions for health, willingness to learn mind-body techniques, and interest in participating in a study of mind-body techniques during MHD. In addition to the survey, data were available on age, gender, race, dialysis vintage, and principal ESRD diagnosis for all study subjects. Data on employment status, educational level, and BMI were available on a majority, but not all research subjects.

### 2.3. Statistical Analysis

Data were analyzed using descriptive statistics for CAM use by demographics including age, sex, race, dialysis vintage, diagnosis of ESRD, and if data were available, employment status, education level, and body mass index (BMI). Individual CAM therapies were grouped into 5 major categories as defined by the National Center for Complementary and Alternative Medicine: (1) biologically-based practices (herbal supplements); (2) manipulative- /body-based practices (chiropractic, massage); (3) mind-body-based practices (yoga, tai chi, qigong, meditation, guided imagery, progressive relaxation, and deep breathing exercises); (4) alternative medical systems (acupuncture, homeopathy, naturopathy, ayurveda, and traditional healers); and (5) energy healing (Reiki). For analyses, we excluded vitamins from biologically based practices since vitamins are usually prescribed as a conventional therapy for MHD patients in the USA. All CAM therapies were combined to a single category to identify any CAM use in a lifetime and the last month. Continuous variables were reported as medians with 25th and 75th interquartile ranges and categorical variables as frequencies and proportions. Categorical variables were compared with Fischer's exact test. The study was approved by the Institutional Review Board at Beth Israel Deaconess Medical Center.

## 3. Results

**Figure 1:**
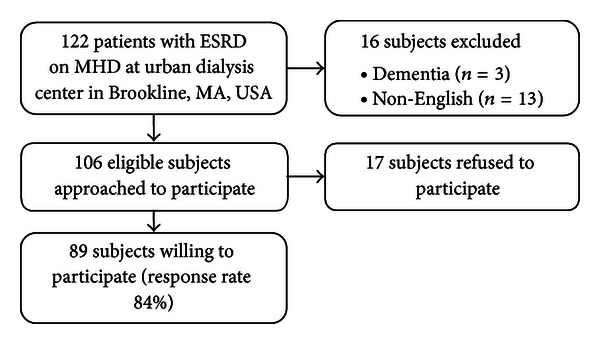
Flow diagram for survey.

Among 106 eligible subjects approached, 89 subjects were willing to participate corresponding to a response rate of 84% (see Figure 1 for flow diagram of survey). Of those subjects excluded from the study, 3 patients had dementia, and 13 patients were non-English speakers. The surveyed sample was 47% female, 63% black, and had a mean age of 62 years. Fifty-four of the patients (61%) reported using CAM anytime in their lifetime (Table 2). The most frequent modalities reported were mind-body practices (used by 42%) and manipulation and body-based practices (used by 34%). The most common mind-body practices reported were deep breathing exercises (27%), meditation (26%), yoga (11%), progressive relaxation (9%), and tai chi (6%).  Table 3 reports patients' characteristics and lifetime use of overall CAM by patient characteristics. CAM use was distributed throughout the population in regards to race, employment status, dialysis vintage, principal diagnosis of ESRD, and BMI. CAM use varied significantly by age as overall CAM (*P* = 0.02), alternative medical systems (*P* = .03), and manipulation and body-based practices (*P* = 0.01) were used most commonly by patients aged between 50 and 64 years (80% overall used CAM) and by only 36% of patients of age 80 years or older. While CAM use was more common in females than males (69% versus 53%, *P* = 0.055) and those with college or higher education than those with high school or less education (79% versus 56%, *P* = 0.075), observed differences did not reach statistical significance. In the last month, 36% of patients reported using some type of CAM. The most frequent CAM modality used in the last month by patients was mind-body practice (27%), while biologically based practices excluding vitamins were used in the last month by 15% of patients.

Subjects reported the perception that mind-body interactions were important to their health with a median score of 9 (interquartile range: 5, 10) on a 10 point Likert scale from 0 to 10.  Table 4 reports the frequency of interest in learning mind-body practices among patients on MHD. A majority of patients reported interest in learning mind-body practices (70%) and participating in a study of intradialysis mind-body practice (74%). Almost half of individuals 80 years or older were interested in learning mind-body practices. Most patients who had used CAM reported interest in participating in a mind-body study during MHD (87%), while only 54% who never used CAM reported interest in participation. The most common reasons for which patients were not interested in learning mind-body practices were lack of interest (*n* = 21), lack of time (*n* = 5), and perception of inability to learn (*n* = 4). 

## 4. Discussion

In our sample population, 61% of patients with ESRD on MHD reported a lifetime use of CAM with 36% reporting use within a month of the survey. The most common modality used by patients in a lifetime or during the last month was mind-body practices. A majority of patients felt that the mind-body connection was very important for their health. An estimated 3 out of 4 patients were interested in learning mind-body practices and participating in research regarding mind-body practices during MHD. 

Limited studies have been conducted identifying patterns of CAM use among patients with chronic disease. National surveys have reported a high overall prevalence of CAM use among patients with arthritis (60%), lung disease (55%), cardiovascular disease (46%), and diabetes (41%) [24]. Prior surveys have reported a wide range of CAM prevalence among patients with ESRD [21–23]. A study conducted in Cincinnati surveyed 153 patients receiving dialysis (both HD and peritoneal) and found 18.3% of patients had used or currently were using CAM [21]. A survey of patients who received renal transplants in Switzerland (*n* = 356) reported 12% prevalence of CAM use [22]. These surveys did not include questions regarding specific mind-body practices such as deep breathing exercises, meditation, yoga, or tai chi. Mind-body practices were the most common CAM modalities we identified in our sample. Comparison of these studies to our own is difficult due to varying definitions of CAM utilized. Our survey represents the first study of CAM use as defined by the National Center for Complementary and Alternative Medicine among patients with ESRD on MHD. 

Our results suggest a positive perception of and willingness to learn mind-body practices among patients on MHD, including during hemodialysis, especially among those patients who have familiarity with CAM. Three small studies have suggested that mind-body practices may be useful for patients with ESRD [25–27]. However, no prior studies have examined the role of mind-body practices during MHD. Conventional exercises (biking and resistance training) have been studied as an adjunctive therapy for patients on MHD [11, 12, 28]; though implementation of exercise programs has been partly low due to poor adherence and limited functional capacity of patients [12]. Mind-body practices are often characterized by low intensity and relaxing effects, which may offer better adherence and be applicable to a broader range of patients on MHD than conventional exercise. In addition, the emphasis of both mental and physical engagements during practice may produce distinct physiological, functional, and psychological changes among patients on MHD. Providing mind-body practices during dialysis may increase awareness and access to patients who have not utilized CAM or mind-body practices in the past. 

About 1 out of 4 patients on MHD were not interested in learning mind-body practices. Only about half of patients of age 80 years and older or those who had never used CAM expressed interest in learning. This is consistent with national data from the general population where fewer elderly patients reported using any CAM or mind-body techniques; only 24% and 10% of individuals of age 85 years and older reported use of CAM and mind-body practices, respectively, in the last 12 months [20]. Potential reasons for less interest are lack of familiarity with CAM modalities, belief that they will be unable to participate, resistance to participating in any additional therapy, lack of time, limited access to CAM therapists or classes, or financial constraints. Further research on perceptions and barriers to CAM therapies among patients with chronic disease and elderly patients is needed to explore these differences and to guide implementation of CAM therapies.

Our study has several limitations. Since our sample population was small and derived from a single dialysis center, our results may not reflect CAM use in other regions or nationally. In comparison to national data of the outpatient dialysis population, our sample had a higher proportion of black patients, while it had a similar distribution in age, sex, and causes of ESRD [29]. Data collected were based on self-report and subject to recall bias. Also, surveys were conducted during MHD among other patients receiving therapy, so subjects may have altered responses due to a lack of privacy. While we utilized standard definitions and questions adapted from nationally based surveys, patients may have a different understanding of what constitutes a CAM therapy. Despite these limitations, our results provide important new insight into CAM use in this chronic disease population.

In summary, CAM use is common among patients with ESRD on MHD, in particular mind-body practices, and is used by a greater percentage of females than males, middle-aged than elderly, and patients with higher education levels. Patients largely expressed interest to learn and participate in studies of mind-body therapies. While CAM use, including mind-body therapies, is frequent in the general population and patients with chronic diseases, there exists limited evidence on their therapeutic efficacy [30]. Further research to develop and evaluate mind-body therapies in patients with ESRD is warranted. 

## Figures and Tables

**Table 1:** Complementary and alternative medicine questionnaire^a^.

Question	Response choices
(i) Have you ever used any of the following for your health? (a) If yes, have you used (CAM modality) in the last month? *Listed options: acupuncture, ayurveda, biofeedback, chelation therapy, and chiropractic care or chiropractor, energy healing therapy or reiki, and folk medicine such as curanderismo or native American healing, hypnosis, massage, and naturopathy *	No, yes, do not know

(i) Have you ever used natural herbs or dietary supplements for your health? For example ginger, Echinacea, or black cohosh (ii) Have you used natural herbs or dietary supplements in the last month?	No, yes

(i) Have you ever used any of the following mind-body techniques for your health? (a) If yes, did you use (CAM modality) in the last month? *Listed options: meditation, guided imagery, progressive relaxation, deep breathing exercises, Yoga, tai Chi andqigong *	No, yes, Do not know

(i) Using a scale from 0 to 10 (where 0 is not at all important and 10 is extremely important) in your opinion, how important are mind-body interactions to your health?	0–10 Likert scale

(i) Would you be interested in learning mind-body techniques at no cost to you?	No, yes

(i) If respondent stated no: Why are you not interested in learning mind-body techniques? *Listed options: i am not interested, mind-body techniques do not work, i do not have time, i won't be able to learn, i donot have the energy, i am too sick, i donot have the money, i do not have a place to learn, and other *	Mark one or more categories

(i) If your doctor agreed, would you be interested in a study that would teach you mind-body techniques that you would practice during dialysis?	No, yes

^a^Adapted from the National Health Interview Survey Adult Complementary and Alternative Medicine Supplement.

**Table 2:** Prevalence of any CAM use and CAM modalities among patients with ESRD on MHD (*n* = 89).

CAM modality	Prevalence (%)
Any CAM use	61
Alternative medical systems	17
Manipulation and body-based practices	30
Biologically based products	14
Mind-body practices	42

**Table 3:** Lifetime prevalence of overall CAM use among patients with ESRD on MHD by patient characteristics (%)^a^.

	No CAM use (*n* = 35)	Any CAM use (*n* = 54)
Percentage of all patients	39	61
Age (years)^b^		
18–34	9	4
35–49	20	15
50–64	20	52
65–79	31	22
≥80	20	7
Female	37	54
Race		
White	29	30
Black	63	63
Other	9	8
Dialysis vintage (years)		
<1	23	30
1–4	34	44
5–9	34	17
≥10	9	9
Principal ESRD diagnosis		
Diabetes mellitus	49	61
Hypertension	20	9
Glomerulonephritis	9	11
HIVAN	6	6
Other	17	13
Employment status	(*n* = 34)	(*n* = 51)
Employed full or part time	12	18
Retired	53	24
Disabled	35	59
Education level	(*n* = 27)	(*n* = 41)
Postgraduate degree	7	12
College degree	4	15
Vocational or high school degree	70	61
Grade school or lower	19	12
BMI ± SD	(*n* = 23) 30.3 ± 10.7	(*n* = 41) 26.3 ± 6.3

CAM: complementary and alternative medicine; ESRD: end-stage renal disease; MHD: maintenance hemodialysis; HIVAN: HIV-associated nephropathy; and BMI ± SD: body mass index ± standard deviation.

^a^Figures are percentages of patients based on the number (*n*) in each group.

^b^Within group differences significant between any CAM use or CAM modality and no CAM use with *P* < 0.05 by Fisher's exact test.

**Table 4:** Interest in learning mind-body practices among patients on MHD (%).

	Interest learning MBP (%)	Interest in participating in study of MBP during MHD (%)
Total (*n* = 89)	70	74
Age (years)		
18–34	100	100
35–49	54	69
50–64	71	74
65–79	78	78
≥80	46	46
Gender		
Female	74	74
Males	66	75
Race		
White	54	65
Black	76	74
Other	71	100
Dialysis Vintage (years)		
<1	75	79
1–4	68	74
5–9	78	89
≥10	50	50
Ever used CAM use		
No	62	54
Yes	74	87

MBP: mind-body practices and MHD: maintenance hemodialysis.
